# Maintenance of Certification – important and to whom?

**DOI:** 10.3402/jchimp.v3i1.20326

**Published:** 2013-04-17

**Authors:** Paul M. Kempen

**Affiliations:** Broadview Heights, OH, USA

In 2000, the American Board of Medical Specialties (ABMS) mandated all 24 specialty affiliates to limit board certification (BC) duration to 10 years. This occurred against initial opposition of several of the affiliates, as the utility, financial, and time impositions, as well as the basic need for such imposition, were openly questioned. Given the threat of losing the ‘franchise’ if not adhering to ABMS corporate mandates, all 24 board affiliates submitted to the ‘10 year policy’ of the ABMS. Subsequently, ABMS recertification programs to renew and maintain BC, now required all physicians to subscribe to increasingly expensive and time-consuming corporate programs, marketed under the name of Maintenance of Certification (MOC). MOC entails yearly and interval consumption of programs, so called ‘licensed products’ of the ABMS affiliates. While MOC continues to be marketed by the ABMS as a ‘voluntary measure’, ABMS has continued pressing strongly for insurance corporations, hospital medical staff, and federal programs to require BC for physician participation and payments, as a ‘Measure of Quality’. As the ABMS itself does not produce educational components, only testing, the MOC educational products have been licensed typically to specialty societies. These national medical societies are eager to earn the significant revenues from these programs – establishing an overwhelmingly powerful academic core industry to propagate the myth of ‘Higher Standards, Better Care’ registered trademark of the ABMS logo and copyrighted ‘Board Certified’ status.

The Federation of State Medical Boards (FSMB) (another ‘testing organization’ producing: FLEX, SPEX, ECFMG, and USMLE) identified corporate market potential and supported this MOC movement developing as early as 2002, a ‘basic’ MOC requirement to practice medicine. The FSMB envisioned coupling MOC as a requirement for state license renewals as their Maintenance of Licensure (MOL) program (1). This regulatory capture effectively insured universal participation (and profits), once adopted. Ohio was the very first state chosen as target state for this FSMB program, with the FSMB Chairman-elect and one additional board member in the highest positions on the State Medical Board of Ohio (SMBO). In October of 2012, over 15,000 Ohio physicians united the 15 Ohio medical organizations (as the first ‘pilot state’ of the FSMB MOL program) to oppose and successfully defeat MOL, the clearly corporate attempt at regulatory capture of the practice of medicine by the FSMB. This also led to the ousting of the Executive Director of the SMBO (2, 3). Thus, state licensing and state medical organizations are removed from the profits of MOC and are best able to rationally identify the realities of MOC for patient care and working physicians. Three national organizations have emerged as leaders in organizing active opposition to MOC and MOL: The American Association of Physicians and Surgeons (http://www.aapsonline.org/), Change board recertification (http://changeboardrecert.com/), and Doctors 4 Patient Care (http://docs4patientcare.org/).

Forty-two years ago, the American Board of Internal Medicine (ABIM) officially endorsed the principle of recertification, but decided to implement it on a voluntary, rather than a mandatory basis. The history of MOC can be traced back to the ABIM's attempts to introduce recertification in internal medicine for those with lifelong certification, via a program exquisitely similar to MOC, then called Continuous Professional Development (CPD) (4). The concept of continuing medical education (CME) was already well entrenched as the American Medical Association's Physician Recognition Award (AMA-PRA) CME documentation program of the late 1960s. Evidence that this CME program was inadequate in assuring the quality of medical care in the USA is/was also lacking, with American medicine's stature as worldwide leader in healthcare. From 1974 to 1986, this ABIM CPD recertification was promoted with notably minimal acceptance, as progressively fewer lifelong diplomats opted to participate in each recertification cycle: 3355 in 1974, 2240 in 1977, 1947 in 1980, and 1403 in 1986 (4). Of particular interest was the finding that certificates of added qualifications, such as geriatric medicine, were to continue to require an active certificate in the underlying discipline (internal medicine). Although the immediately Ex-President of the ABIM was a member of the same 2000 ABIM Task Force on Recertification and originally certified in medicine in 1979, (re-) certification in geriatrics occurred only in 1998 and 2005, *without the recommended primary recertification in internal medicine*. Similarly, another ABIM board member's testimonial to the value of MOC and recertification, openly documented his complete failure to recertify in medicine while leaving a 25-year certification interval in oncology prior to recertification in 2008. His reasoning: ‘So why did I recertify? The truth is, I had to. As a member of the ABIM Board of Directors, I am required to participate in MOC as a condition of my service.’ (5) This reasoning is no different to the testimonial from the new Chairman of the ABIM (who originally certified in 1986, and only recertified in 2009 prior to assuming the ABIM chairmanship) in his online blog (6). The blog is well worth reading, as the clear, rational responses of frustrated physicians overwhelmingly reject MOC, whether certification is time limited or not. Dr. Nora, the new ABMS CEO, certified only once in 1987 as did Dr. Chaudhry, the FSMB CEO, in 1996 and neither recertified or enrolled in MOC, while leading respective organizations to impose MOC via regulatory capture. The participation patterns of these leaders specifically raise significant questions as to any personal vs. *corporate* value to these programs. The rejection of elective recertification was widely documented by a survey performed in the *New England Journal of Medicine* as recently as 2010, where clearly two thirds of all physicians overwhelmingly rejected MOC for physicians with lifelong certification (7, 8).

So why comply with MOC? The evidence now presented in this journal by Buscemi et al. (9) is particularly timely and of great value. We again have overwhelming and contemporary documentation of the fact that BC renewal has a very long history of being rejected, not only lacking value in insuring one remains ‘up to date’ by the general population of practicing physicians, but also *by the very individuals governing the corporations selling MOC*. It is increasingly evident that these board members value BC, much like the population in general, using BC specifically as a means to market themselves, to secure the next (or maintain the current) employment position and to exclude non-certified competition from the market place. While Buscemi et al. studied the local relationships in practicing physicians in Texas (where the FSMB is centered), their findings are reflective of the national reality. Clearly, the review of the ABIM Board of Directors’ participation patterns and positions, confirms MOC as a simple ‘product to be marketed to the masses’. This comes as no surprise: We can be readily reassured that the Board of Directors of McDonalds or Burger King do not routinely gorge themselves on hamburgers, either. These board members of the certification industrial complex (CIC) have the primary fiduciary responsibility to promote the welfare of the corporations and their profits, in this increasingly difficult era of cost containment. As leaders, they line up to ‘comply’ with the corporate employment requirement and not out of any deep-seated moral conviction. It is important for all physicians to recognize the clear corporate nature of recertification: BC originated as a means to establish national outcome criteria for excellence in residency training programs. It became a ‘right of passage’ and served to enhance training primarily via the goal of specialist education beyond simple ‘on the job training’. Historically, ‘passing rates’ were comparatively evaluated to judge individual training program's capabilities, when no national oversight existed. Now national accreditation of residency training is a reality, and the importance of recertification is being denigrated from ‘specialist excellence’ to a basic licensure requirement – mandated mainly for private corporate profit.

Buscemi et al. correctly identify that there is no significant supportive documentation in world literature that MOC, or even BC itself, is proven to result in the ‘Higher Standards, Better Care’ promised by the ABMS logo. Certainly, multiple publications based on retrospective chart reviews, typically coauthored by employees of this ABMS/FSMB CIC, *are offered by the industry itself*, to document associations of positive effects from MOC and BC. Given the inherent conflict of interest of these corporate authors, the validity of the basic assumptions, statistics, and methods precludes anything beyond a need to perform randomized trials to evaluate the ‘noted association’, in this era of ‘outcome based medicine’. The concept of retesting ‘basic textbook knowledge’ in closed examinations, when online referencing is standard practice, as opposed to promoting ongoing specialization and updates on new medical horizons, seems paradox, with the increasing delegation of non-physician ‘providers’ (i.e., nurse practitioners, physician assistants, CRNAs, etc.) to function competitively alongside physicians without oversight.

The imposition of practice improvement modules often requires the MOC participant to document care and then change it ‘for the better’. Patients become research subjects paying for any new prescriptions, now for national recertification purposes, without their knowledge or consent, solely to meet physician certification needs. Imposing medical care for purposes other than an individual patient's benefit was overwhelmingly rejected by the Nuremberg code of 1947, which stressed the importance of patient informed consent, after national ‘quality improvement programs’ strived to improve the genetic makeup of that nation. It would be clearly unacceptable to allow, for example, hypoglycemic episodes (and possibly death) to result in any individual, simply to improve (lower) practice averages in HbA1c for recertification Practice Improvement Modules (PIM) purposes. The following testimonial of PIM ‘success’ is, for example, completely medically irrelevant: ‘I was able to identify some gaps. For example, I chose to assess how often informed consent forms actually made it into the electronic medical records (EMRs)’ (3). Just what does this have to do with medicine or quality? Simulation has also been introduced as a basic MOC module requirement in anesthesiology at great cost. Simulation was repeatedly never validated as a tool to impart long-term retention of medical motor skills vs. knowledge (the specific thrust of simulation), in Advanced Cardiac Life Support (ACLS) scenarios (see added references). Simulation is merely one educational tool for the introduction of new ideas to students, or rare events to experienced individuals, while avoiding patient endangerment. Rare events remain exceedingly difficult to study clinically, making clinical validation of simulation directed at such scenarios difficult to impossible.

Every patient is a test in the practice of medicine. Oversight of physicians is already and increasingly imposed by hospital administrations, patients, families, colleagues, private and federal insurance as well as malpractice carriers, police, The Joint Commission, DEA, State Medical Boards, and attorney general, the Department of Health and Human Services, to name a few. Do we need to have another private and corporate oversight agency imposing yet another fiscal burden on the overregulated physicians and healthcare? The MOC program is especially demanding of independent, rural, primary care providers, who must travel to distant sites to absolve the multitude of obligational programs of MOC. CME expenditures reached over $ 2.5 billion dollars in 2007 and the gross receipts of the ABMS/FSMB CIC in 2009 reached $ 350 million (10). These sums noted are *only* for the registration participation fees, without the 1–2 times greater expenditures for travel, housing, and, yes, locums cross coverage! This documents the significant physician commitment to ‘lifelong learning’!

Board certified is a past tense connotation. Every physician should be encouraged to become certified early after residency, once and for life, and undergo lifelong learning personally tailored to his practice and patient population. Only the physician himself can determine the means and goals to his personal needs in patient care – not some ‘one size fits all’ MOC program. MOC is neither a proven concept nor a single product and recently has been questioned as ‘the answer to lifelong learning (11). MOC programs are ‘works in progress’, being currently developed by each individual ABMS board for each individual specialty certification. The many studies reported by the ABMS have very limited scientific validity, as determined by ABMS's own sponsored and authored analysis, in 2002. This attempt at meta-analysis concluded less than 5% of all prior studies attempting to demonstrate the value of BC ‘used research methods appropriate for the research question’. The remaining studies were additionally not qualitatively amenable to support meta-analysis (12). As such, the physician population is also subject to ABMS ongoing research and without majority consent. The ABMS protocol also requires extensive signed release of multiple rights and legal recourse, freeing ABMS of liability, to simply participate in the ABMS programs (13). If the ABMS wishes to market their products in the CME marketplace, using an atmosphere of competition with all CME providers, that would be welcome. This is *not* occurring under time-limited BC or MOL. The ABMS has already lobbied congress to nationally couple physician payments to MOC under the Physician Quality Recognition Payment–MOC program. Incentives become penalties after 2014, imposing effective regulatory capture of the medical profession into MOC (10, 11). In this Internet era, programs designed to facilitate education from home will provide value to patients, physicians, and medicine at large. Continued marketing of time-limited certificates will lead to increasing numbers of physicians either practicing with ‘expired’ certification or retiring early, if ‘economic credentialing’ occurs from this ABMS/FSMB plan. Certification for life is, and has been, the ‘real deal’. There are no published studies comparing physicians who have completed the MOC program and those who have not. There is however medical excellence practiced in Canada and Europe, where the ABMS and FSMB have *never* certified anyone.

The MOC premise that physicians become outdated with extended time in practice in all specialties is a fallacy. Experience is the significant factor at multiple levels of medical decision. One cannot treat what one does not recognize. Referral is the ultimate answer, when recognizing the limitations of one's own care, or that provided locally. Tertiary centers with the complete range of options and subspecialists are often the only place some patients will receive appropriate care. ‘A Man has to know his limitations’ – Harry Callahan. This is extremely important in medicine also, *as one will never know it all*. Certification is at best a slight, or possibly false, promise, recently openly admitted by the ABMS: ‘FACT: *ABMS recognizes that regardless of the profession – whether it is health care, law enforcement, education or accounting – there is no certification that guarantees performance or positive outcomes*’.
